# The psychosocial impact of migraines on women and alternative therapies for migraine management

**DOI:** 10.4102/hsag.v28i0.2249

**Published:** 2023-09-13

**Authors:** Ashalya Pirthiraj, Raisuyah Bhagwan

**Affiliations:** 1 Department of Chiropractic, Faculty of Health Sciences, Durban University of Technology, Durban, South Africa; 2 Department of Community Health Studies, Faculty of Health Science, Durban University of Technology, Durban, South Africa

**Keywords:** migraines, female migraineurs, psychological impact, social impact, psychosocial factors, chiropractic, complementary therapies, alternative therapies

## Abstract

**Background:**

Migraines are one of the leading causes of disability globally and in South Africa. There is a paucity of local empirical literature regarding the psychosocial impact of migraines on women. Although there are a variety of treatment approaches, many women prefer alternative and holistic treatment for their migraines.

**Aim:**

The aim of this study was to explore the psychosocial impact of migraines on women and their use of complementary and alternative therapies for migraine pain management.

**Setting:**

The study was conducted in the eThekwini region of KwaZulu-Natal, South Africa.

**Methods:**

The study adopted a qualitative descriptive design. Purposive sampling was used to recruit participants for the study. Data were collected through 12 semi-structured interviews and subsequently analysed using thematic analysis.

**Results:**

Theme 1 related to the psychological and cognitive effects experienced by the participants. Theme 2 focused on the effects migraines had on personal, family and social relationships. Theme 3 discussed the self-management of migraines.

**Conclusion:**

The pertinent psychological effects of migraines were depression, anxiety, feelings of hopelessness and withdrawal, fear-avoidance behaviour, lifestyle changes, and acceptance of migraines. The participants experienced a lack of understanding about their migraine severity from co-workers, family and social networks. The alternative therapies that were sought to alleviate migraine symptoms included chiropractic, massage, meditation, reflexology, yoga, cupping and acupuncture.

**Contribution:**

The awareness of the effectiveness of complementary and alternative therapies for women may be beneficial for healthcare providers seeking a multidisciplinary approach to migraine management.

## Introduction

There is a high global burden of disease (GBD) associated with migraine headaches. The study in 2015, declared migraines as the third leading cause of disability worldwide for those under the age of 50, and in the GBD study in 2010, it was the third most prevalent disorder in the world (Steiner, Stovner & Vos 2016:104). Among urban hospital outpatients in South Africa, migraines contributed to severe psychological distress in 39.2% women and 23.5% men (Peltzer, Pengpid & Skaal 2012:10).

Migraines are recognised by the World Health Organization (WHO) as being among the world’s leading causes of headache-related disability. Headache-related disability is dependent on the frequency and severity of the migraines. The Migraine Disability Assessment (MIDAS), which is used to measure headache-related disability, evaluates days of missed activity or markedly reduced activity in the preceding 3 months for work; school; productivity at work; not completing household chores; productivity in household chores; and leisure, family, or social activities (Dodick et al. 2016:826). Gibbs et al. (2020:1352) listed some of the disabling effects of migraines such as decreased concentration, daily activity impairment, reduced productivity and missed workdays. A comprehensive study by Estave et al. (2021:1004) reported that migraineurs’ – that is, people who experience migraines – indicated that their migraines caused disability during attacks, they experienced a lack of control and they attempted to push through it despite their pain.

Migraines are predominantly experienced by women, with the peak prevalence between 25 and 55 years of age (Truter 2015:448):

Migraine is two to three times more prevalent in women than men, and women report a longer attack duration, increased risk of headache recurrence, greater disability, and a longer period of time required to recover. (Vetvik & MacGregor 2017:76)

Hormonal fluctuations play a role in precipitating migraine attacks. Another study suggested that the drop in oestrogen levels may trigger migraines before or during menarche (the onset of menstruation), as well as during the postpartum period, and the perimenopausal or postmenopausal period (Verhaak et al. 2021:70). Women may experience migraines before or during menstruation, known as menstrual migraine (Vetvik & MacGregor 2017:83). Menstrual migraine without aura is most prevalent, and it may be associated with a longer duration of migraines (International Headache Society 2018:19). Migraine with aura occurs in approximately 20% of migraineurs and precedes the migraine. The aura can be associated with a range of temporary visual symptoms, sensory symptoms, brainstem symptoms, or motor symptoms (International Headache Society 2018:19).

Migraines have a significant psychosocial impact. Several international studies have documented that migraineurs feel misunderstood, isolated, frustrated, and hopeless about their inability to manage their migraines. In addition, they indicate that migraineurs experience an associated decrease in their quality of life, and a negative social impact in the workplace, as well as in their personal and family relationships (Befus et al. 2019:3; Gibbs et al. 2020:1352; Leonardi & Raggi 2019:6; Palacios-Ceña et al. 2017:9; Persson et al. 2021:8). Furthermore, the impact on their emotional health included anxiety, depression, frustration, anger, irritability, mood changes, hopelessness, feeling isolated, and concentration difficulties (Estave et al. 2021:1004). Malone, Bhowmick and Wachholtz (2015:539) highlighted that 71% of their study participants encountered disbelief regarding the severity of their migraines from those in their social networks. There is a paucity of international and local empirical studies in South Africa that highlight the psychosocial burden on female migraineurs. The available literature revealed that South African university students neglected family or social activities, and experienced reduced concentration and an inability to complete tasks or daily activities without assistance during migraine episodes. Hence, they felt as if they were a burden to others (Basdav, Haffejee & Puckree 2016:1680).

There is a high socio-economic burden associated with migraines. It is an expensive condition to treat in South Africa, as healthcare systems fail to provide effective treatment for migraines, and this consequently results in decreased productivity in the socio-economic sector (Truter 2015:450). Migraines are not always diagnosed or treated and most sufferers have had delays in diagnosis, inadequate treatment, and the resultant feelings of helplessness and depression (Banciu & Bouleanu 2018:29; Persson et al. 2021:8). Migraineurs reported dissatisfactory or insufficient treatment, despite the variety of treatment approaches that are available (Malone et al. 2015:537). Migraineurs’ employers are also affected by absenteeism (absent from work because of a migraine), presenteeism (at work with a migraine), reduced individual productivity and consequently the burden of reduced economic productivity (Gibbs et al. 2020:1356). In a survey of 70% female participants, 22% of work time was missed because of absenteeism, 60% of work time was impaired because of presenteeism, and 65% was attributed to work productivity loss caused by migraines (Gibbs et al. 2020:1356). Vo et al. (2018:326) suggested in their study that presenteeism contributed to work productivity loss and impaired activity. Furthermore, migraineurs (majority were female) were reported to have lost 6 h from work on average in a week because of problems associated with their migraines (Gibbs et al. 2020:1356).

Natural, alternative, or holistic approaches to health are often sought by migraineurs. There are many complementary treatment options available for migraineurs. Complementary and alternative medicine (CAM) are therapies that are considered complementary or adjunctive to conventional medicine. Complementary and alternative medicine therapies for migraine treatment resulted in a 27% decrease in moderate mental distress in women (Rhee & Harris 2017:97). Women use CAM more frequently than men (Rhee & Harris 2017). A study conducted in the United States, with a predominantly female population (83.33%), explored the non-pharmacological self-management of migraines, and migraineurs’ desires and recommendations for migraine clinicians. Four themes were identified: ‘a more holistic, collaborative, long-term treatment approach; medication as a short-term solution; high personal and economic costs of medication; and desire for more information and access to natural approaches’. The study also found that migraineurs expressed hopefulness for potential migraine relief with alternatives to medication (Befus et al. 2019:4).

‘One promising non-pharmacological approach for migraine is chiropractic care, due to the co-occurrence of migraine disease and musculoskeletal tension and pain’ (Wayne et al. 2020:1). In essence, Chiropractic is:

A health profession concerned with the diagnosis, treatment and prevention of mechanical disorders of the musculoskeletal system, and the effects of these disorders on the function of the nervous system and general health. There is an emphasis on manual treatments including spinal adjustment and other joint and soft-tissue manipulation. (World Federation of Chiropractic 2001:1)

Chiropractors are primary healthcare physicians who use a manual therapy technique called ‘manipulation’ or ‘adjustments’. This is a high velocity-low amplitude (HVLA) manual thrust manoeuvre that is directed to specific spinal facet joints (spinal manipulation) or extremity joints (extremity manipulation) (Rist et al. 2019:533), for the purpose of increasing range of motion and stimulating the nervous system. A comprehensive study indicated that ‘research has suggested that spinal manipulative therapy (SMT) may stimulate neural inhibitory systems at different spinal cord levels and might activate various central descending inhibitory pathways’ (Chaibi et al. 2017). Thus, the hypoalgesic effect (reduced sensitivity to painful stimuli) of SMT may contribute to the decreased pain levels in migraineurs receiving chiropractic treatment. Chiropractors address the musculoskeletal complaints associated with migraines and treatment has been shown to reduce the intensity and duration of migraines (Chopdat 2015:vii; Rist 2019:532).

Knowledge of migraine patients’ experiences can contribute to better doctor–patient communication and compliance with treatments (Palacios-Ceña et al. 2017:11). There is a need for a more in-depth understanding of the difficulties experienced with migraines and the accompanying impairments that manifest in social activities or work (Banciu & Bouleanu 2018). Migraines have a significant impact on one’s lifestyle; it changes the individuals’ lived experience, increases their vulnerability, and is accompanied by severe pain; and various attempts are made to cope with migraine-related pain, and low expectations of healing are common among migraineurs (Banciu & Bouleanu 2018:29). This highlights the importance of understanding coping mechanisms and treatment approaches to migraine management.

## Aim of the study

The purpose of the study was to explore the psychosocial impact of migraines on women and their use of complementary and alternative therapies for migraine pain management.

## Methods

### Study design

This was a qualitative study that used a descriptive design. It was a descriptive study in that it sought to describe the psychosocial impact of migraine headaches on women and their use of alternative therapies to address the latter. Qualitative descriptive studies ‘provide rich descriptive content from the subjects’ perspective’ (Colorafi & Evens 2016:24). Furthermore, qualitative descriptive studies are applicable to healthcare environments as they describe participants’ emotions, reasons for using healthcare services, and the factors that facilitate its use (Colorafi & Evans 2016).

### Setting

The study was conducted in the eThekwini region of KwaZulu-Natal, South Africa. Face-to-face and virtual video call interviews were used for data collection to conform to COVID-19 pandemic regulations. At the time, institutional approval for the study was sought, face-to-face interviews were restricted, and physical distancing and wearing of face masks were mandatory. The researcher and participants were therefore required to maintain a safe distance, wear face masks, and thereby adhere to the necessary COVID-19 protocols. As the COVID-19 pandemic influenced the process of face-to-face interviews, video call interviews were also used for data collection. For the face-to-face interviews, the researcher liaised with the participants to confirm the most accessible and convenient venues to conduct the interviews. Prior studies conducted in KwaZulu-Natal quantitatively documented literature pertaining to migraineurs (Basdav 2016; Du Preez 2004; Kleingeld 2016). However, no studies were undertaken to qualitatively report the experiences of migraineurs in this region.

### Study population and sampling strategy

This study was conducted with females between the ages of 18 and 65 who presented to chiropractic practices, for migraine treatment in the eThekwini region of KwaZulu-Natal. Purposive sampling was used to recruit participants for the study to obtain information-rich data.

Inclusion criteria were as follows:

- Adult females between the ages of 18 and 65;
- Women with acute, episodic, or chronic episodes of migraine headaches with or without aura;
- Participants who were patients of chiropractors and had received chiropractic treatment for migraines;
- Those who consented to participate in the study.

### Data collection

The main researcher liaised with the participants who were recruited for the study to determine if they were willing to have face-to-face interviews conducted with adherence to the COVID-19 safety protocols. Online video call interviews were primarily used for data collection. Data were collected qualitatively through 12 semi-structured interviews, which lasted approximately 45 min, using an interview schedule. Permission was obtained from each participant to voice record the online and face-to-face interviews. After data were collected from 12 participants, saturation was reached as information-rich data were collected. Eleven interviews were conducted online, and one interview was conducted face to face. Data collection was completed between March 2021 and September 2021.

The interview schedule consisted of the following questions:

- Can you describe your personal experiences of migraines?
- How does this experience affect your relationships with others?
- How do you feel during a migraine episode? Probes: Family life, work, impact on life.
- Can you describe some of the social consequences of getting migraines? Probes: Social events.
- What do you do to cope with the migraine pain?
- What alternative therapies have you used to help you cope with your migraines?

### Trustworthiness

The model by Lincoln and Guba was used to achieve rigour and enhance the trustworthiness of the study (Nowell et al. 2017:3). Strategies used to ensure credibility included member checking and theoretical triangulation. The research reflected the views of the participants as accurately as possible, thereby ensuring credibility. For verification, the participants’ statements were checked and confirmed by the participants. To ensure transferability, the research process and results of the study were thoroughly detailed, and sufficient information about the study was provided for it to be generalised or transferred to other contexts. To ensure dependability, the research process was clearly documented and traceable for it to be replicated. The study achieved confirmability by creating an audit trail that detailed the process of data collection, data analysis and interpretation of the data. Reflexivity was achieved by keeping a research diary to record the thoughts and observations during data collection and analysis.

### Ethical considerations

The study was approved by the Institutional Research Ethics Committee (IREC) in the Faculty of Health Sciences at the Durban University of Technology (IREC no. 173/20). Permission to recruit participants from chiropractic practices in eThekwini, KwaZulu-Natal, was obtained. Consent to participate in the study was secured using a letter of information and informed consent form, which was provided to each participant to read and sign before the commencement of each interview. The participants were required to provide informed consent via email prior to the online interviews. The letter of information and informed consent form described the pertinent information regarding the study and included the information required from the participant and their involvement in the study. Participation was voluntary, and the participants were informed that they could withdraw from the study at any time. Confidentiality was upheld by storing all electronic data on the main researcher’s password-protected laptop. In addition, all hard copies were stored in a locked cabinet at the main researcher’s residence. Upon completion of the study, all electronic data and hard copies were stored in a locked steel cabinet in the Chiropractic Department at the Durban University of Technology. All data will be disposed of in an appropriate manner after 5 years.

### Data analysis

The data were analysed using thematic analysis. Thematic analysis is used to identify and analyse patterns in qualitative data (Clarke & Braun 2013:2). The interviews were recorded using an audio-recorder and were then transcribed verbatim by the researcher. The transcripts and recordings were cross-checked multiple times to ensure accuracy of the data. A second coder was used to eliminate researcher bias. The data were analysed by finding similarities in the transcripts, which led to the formation of sub-themes that emerged from the main themes.

The six phases of thematic analysis, as proposed by Clarke and Braun (2013:3), were utilised to analyse the study’s data. During the first phase, which was ‘familiarisation of data’, the main researcher ensured that accurate data capturing took place by repetitively listening to the audio recordings. Similarities across the transcripts were noted when transcribing each interview. The second phase comprised the process of ‘coding’. The questions from the interview schedule were used to guide the formation of labels for relevant information from the transcripts, and thereafter codes were generated. The third phase entailed ‘searching for themes’. The similarities and patterns within the data were used to construct the main themes. Codes were thereafter assigned to the themes and sub-themes were formulated. The fourth phase ‘reviewed the themes’. The main themes were reflected upon to make sure that they were relevant to the research questions and objectives of the study. The codes under the themes and sub-themes were reviewed to ensure that they reflected relevant meaning to the study. The fifth phase included ‘defining and naming the themes’. Concise names for the themes and sub-themes were constructed by the identification of the essence of each theme. Each theme is broadly related to its sub-themes. The sixth phase involved producing the report by ‘writing up’ the results of the analysis. Relevant information was extracted from the transcripts for the themes and discussed in relation to the existing literature.

## Results

Table 1 depicts the demographic profile of the participants, particularly their age at the time of the study and their occupation. Table 2 presents the migraine history of the participants, especially the age of onset of the migraines and if they experienced episodic migraines or chronic migraines. Migraines that occur for less than 15 days per month are classified as episodic migraines. Migraines that occur for more than 15 days per month, and for more than 3 months are classified as chronic migraines (International Headache Society 2018:24). Table 3 includes a summary of all the themes and sub-themes. Theme 1 (Box 1) related to the psychological and cognitive effects experienced by the participants. The sub-themes that emerged were depression, anxiety, feelings of hopelessness and withdrawal, fear-avoidance behaviour and lifestyle changes, and acceptance of migraines. Theme 2 (Box 2) focused on the effects of migraines on relationships. The sub-themes that emerged were the impact on family and social networks, which included both strained and supportive relationships, and the impact on work life. Theme 3 (Box 3) related to the self-management of migraines. The sub-themes that emerged were resistance to medication, factors that alleviated migraines and alternative therapies.

**TABLE 1:** Demographic profile of the participants.

Participant	Age	Occupation
1	32	Sales representative
2	31	Internal sales
3	27	Business development officer
4	30	Quantity surveyor
5	38	Police official
6	25	Electrical engineer
7	44	Operations manager
8	51	Housewife
9	62	Housewife
10	45	Personal trainer
11	40	Hairdresser, beautician, teacher
12	29	Merchandising manager

**TABLE 2:** Migraine history of the participants.

Participant	Age of onset of migraines	Episodic or chronic migraine
1	18	Chronic
2	20	Chronic
3	19	Chronic
4	18	Episodic. At age of onset – chronic
5	21	Chronic
6	22	Chronic
7	14	Chronic
8	19	Chronic
9	18	Episodic. From the age of onset until menopause – chronic
10	Teenager (between 13 and 17 years of age)	Chronic
11	Puberty (between 8 and 13 years of age)	Episodic
12	10	Chronic

**TABLE 3:** Themes and sub-themes.

Theme	Sub-themes
1. Psychological and cognitive effects	1.1 Depression and anxiety 1.2 Feelings of hopelessness and withdrawal 1.3 Fear-avoidance behaviour and lifestyle changes 1.4 Acceptance of migraines
2. Effects on relationships	2.1 Impact on family and social networks: From strained to supportive relationships 2.2 Impact on work life
3. Self-management of migraines	3.1 Resistance to medication 3.2 Alleviating factors and alternative therapies

BOX 1 Theme 1, sub-themes and excerpts.

**Table:** 

Theme One: Psychological and cognitive effects
**Sub-theme 1.1: Depression and anxiety:** ‘You get really depressed. You have anxiety. You always feel like you’re a sickly person.’ (Pt 1) ‘… you’ll get depressed, or worried because you’re constantly having these headaches. It becomes so frustrating.’ (Pt 2) ‘I have anxiety medication that I take. Sometimes it gets so bad that you actually do want to die.’ (Pt 3) ‘Lots of anger as well, like why do I have to go through this and why does it have to be so painful?’ (Pt 8)
**Sub-theme 1.2: Feelings of hopelessness and withdrawal:** ‘I felt like such a liability. The mood swings are intense. I close off and I don’t really want to speak to anyone.’ (Pt 4) ‘You become more introverted. You do generally withdraw, become more emotional, and very sensitive.’ (Pt 1) ‘You don’t want to be around people that don’t understand you. I limited how much people I was in contact with just to eliminate the stress that it may have caused.’ (Pt 6)
**Sub-theme 1.3: Fear-avoidance behaviour and lifestyle changes:** ‘Now I have to be extra careful. It affects my daily life. I don’t watch 3D or 4D or 5D movies, no clubbing, and no driving at night because of the oncoming cars lights. You have to take precautions to not trigger it.’ (Pt 3) ‘It was a massive fear and it still is to this day when I have any public speaking or meetings with clients, just very scared when I’m meeting new people and should an aura happen with them.’ (Pt 4) ‘I have to keep in mind of not drinking certain things and not staying out too late because I don’t want to jeopardise not getting enough sleep and then waking up with a migraine. If I go out with friends, I can’t eat or drink certain things. I always have to be aware of everything like I can’t just do whatever, whenever. I always have to be careful that it’s going to come on.’ (Pt 12)
**Sub-theme 1.4: Acceptance of migraines:** ‘I’ve accepted it. There’s no point in stressing about it because it is what it is. I’ve accepted that there’s a lot I can do to change it.’ (Pt 1) ‘Life continues. My daily routine has to continue. Everyone’s accepted that this is the problem and I’m going to have it for the rest of my life. There’s nothing you can do about it.’ (Pt 5) ‘I get used to it. I just ignore whatever symptoms I’m having. It’s not going away. I can’t really let it affect me. It is affecting me, but I just have to carry on. I can’t just lie down and sleep. There are things to do and kids to see to. I don’t like to feel like a sick woman because it’s very debilitating.’ (Pt 7) Interestingly, one participant could not accept the disability associated with migraines: ‘I’ve got it for decades. It’s something that I just cannot accept, although it’s around for so long. It’s supposed to be part of my life. I’m supposed to be accepting it. I cannot handle the drama it puts me through.’ (Pt 8)

Pt, Participant.

BOX 2 Theme 2, sub-themes and excerpts.

**Table:** 

Theme 2: Effects on relationships
**Sub-theme 2.1: Impact on family and social networks: From strained to supportive relationships:** The following excerpts reveal the impact of migraines on family life: ‘In the beginning it was very difficult for my family to understand, especially my mother. It’s very difficult for your mum to understand that she can’t be there all the time.’ (Pt 1) ‘In the past, my husband hasn’t been exactly very understanding. Sometimes it’s like you’re saying you have a headache because you don’t want to be there and you want to use that as an excuse to come home.’ (Pt 7) ‘If you’re living with other people you’ve got to get up and cater for them. You can’t even rest.’ (Pt 9) The following excerpts reveal the impact on social life: ‘From a social point of view, there was never living your life and having any sort of social interactions after that. Socially it’s still pretty difficult to explain why you’re not getting a second drink or why you’re not drinking at all. When you’re going out with work colleagues, you’re drinking. So from that point of view, it was very difficult.’ (Pt 4) ‘It interrupts my social life if I make commitments and I’m not well, I’ve had to cancel. I need to be around people that I can trust. I can’t afford for people to judge me or get upset with me because I can’t make a function because I’m not well.’ (Pt 1) ‘To avoid answering questions, I used to stay away from social functions.’ (Pt 9) ‘My social life has taken a hit, but I found a new group of friends who also have migraines, so they understand.’ (Pt 3) Some participants experienced a lack of understanding from others, as reflected in the excerpts below: ‘Everybody is like, it’s just a headache. They don’t understand.’ (Pt 8) ‘The only person who’d actually sympathise or know what you’re going through is another migraine sufferer, and at home, sometimes you feel like you’re just complaining, and they wouldn’t understand. Sometimes I won’t say I’ve got a headache.’ (Pt 7) ‘It was such an invisible illness that I’ve had people over the years say, just take a Panado, come out with us, you’ll be fine. That’s not how it works.’ (Pt 4) ‘Nobody knows the pain that you go through unless they go through it themselves.’ (Pt 9)
**Sub-theme 2.2: Impact on work life:** ‘Previous managers were not sympathetic. Not everybody at work understands that it’s not just a bad headache. There are some days when I just have to call in sick for a migraine.’ (Pt 3) ‘It’s not always easy to say I’m not well again.’ (Pt 1) ‘It creates more pressure on you because you constantly have that headache there. You’re still getting all your work done and it does stress you out a bit more.’ (Pt 2) ‘I just work through it because it’s part of everyday for me now. With work I can’t afford to take time off because it’s there. I ignore it.’ (Pt 7) ‘Definitely it does negatively affect my work in terms of not being able to be at work all the time.’ (Pt 12) ‘They know it’s serious and they’re very understanding about it luckily.’ (Pt 4)

Pt, Participant.

BOX 3 Theme 3, sub-themes and excerpts.

**Table:** 

Theme Three: Self-management of migraines
**Sub-theme 3.1: Resistance to medication:** The following participants were resistant to pharmacological treatment: ‘This medication has helped, but it’s not something I want to be on. It’s not good for you. If I can do things holistically and change my lifestyle as best as I can to improve my situation, then I will.’ (Pt 1) ‘I try not to take medication, because I feel it takes such a long time to get out of your system.’ (Pt 4) ‘The doctor did put me on chronic medication, but I stopped taking that years ago. I do not want to take it because it has side effects.’ (Pt 5) ‘I can’t be taking tablets every day and the pain is quite severe. I said to the doctor, I rather use something naturally than taking medication.’ (Pt 7)
**Sub-theme 3.2: Alleviating factors and alternative therapies:** The following excerpts reveal the alleviating factors that participants found beneficial: ‘Cold showers and ice packs. I’ve even tried Botox at the back of my neck. It does help but it’s very short-term relief. So, for me it’s a very expensive form of relief.’ (Pt 1) ‘I do stretches, I bought a pillow, I changed my bed. I change my posture.’ (Pt 3) ‘I have tried mainly medication and injections and the injections worked very quickly to ease my headache, but it didn’t really help me. It helped me for that time only, or it came back full force after.’ (Pt 6) ‘I started doing more strength training and weight training, and I found that it’s helped me so much.’ (Pt 4) ‘I take a migraine pack and I just put something heavy on my head and cover my eyes.’ (Pt 8) ‘I drink water and get some sleep.’ (Pt 12) The following excerpts reveal the alternative therapies that participants found beneficial: ‘I go for massages. Acupuncture and seeing a chiropractor helps, and it’s definitely more manageable.’ (Pt 1) ‘It’s chiropractor first, then supportive pharmaceutical medication that helps break the migraine.’ (Pt 3) ‘The only thing that helped me was going to the chiropractor.’ (Pt 6) ‘Reflexology actually helped a little bit.’ (Pt 4) ‘I’ve done cupping. It was temporary relief.’ (Pt 2) ‘Apart from the massaging and exercise, I do yoga and meditation.’ (Pt 7)

Pt, Participant.

## Discussion

Theme 1 related to the psychological and cognitive effects experienced by the participants. Four sub-themes emerged from the data analysis. The first sub-theme is ‘depression and anxiety’. Migraineurs are known to experience depression three times more commonly than those without migraines (Dindo et al. 2015:109). Furthermore, the study indicated that the comorbidity of migraines with depression and anxiety is highly significant as it has an increased risk for disability, medication overuse, and suicide (Dindo et al. 2015:109). In this study, half of the participants experienced anxiety and depression. Furthermore, feelings of sadness, worry, anger and frustration were highlighted by the participants. Feelings of sadness, frustration and vulnerability were also experienced by women with migraine without aura in a qualitative study that was conducted by Banciu and Bouleanu (2018:27). In addition, other chronic migraine sufferers also felt anger and irritability about the frequency of migraine attacks (Banciu & Bouleanu 2018:28; Persson et al. 2021:8). Suicidal thoughts during severe migraine attacks were experienced by chronic migraine sufferers (Persson et al. 2021:8). Similarly, suicidal thoughts were experienced by one participant because of the migraine severity. Anxiety and depression were common across all migraine frequency subgroups (episodic and chronic migraineurs) in a cross-sectional study undertaken in 17 European countries (Vo et al. 2018:326).

The second sub-theme of Theme 1 related to ‘feelings of hopelessness and withdrawal’. Participants in this study felt hopeless, irritable, and were short-tempered, and emotional when experiencing migraines. They expressed a need to withdraw from people until the migraine passed. One participant stated that she was obliged to interact with family while experiencing migraines, which led to irritability. Some participants felt as if they were a burden and liability to those around them, especially to their families. The literature revealed that migraineurs experienced an emotional impact such as hopelessness, irritability, despair, frustration, anger, guilt, feeling like a burden, a lack of control, mood changes, and feeling isolated from others (Banciu & Bouleanu 2018:28; Basdav et al. 2016:1680; Mannix et al. 2016:6; Palacios-Ceña et al. 2017:9). Women with migraines without aura were reported to have experienced helplessness and frustration because of the unpredictability, invisibility and permanence of their migraines (Banciu & Bouleanu 2018). Female migraineurs also felt hopeless for the inability to manage their migraine pain (Persson et al. 2021:8). Two participants reported feeling insecure and hopeless about their ability to be independent in their lives because of a history of relying on others for assistance during their migraine attacks.

Migraines disrupt daily activities, including women’s ability to perform household chores as well as taking care of themselves or those dependent on them, such as their children. The temporary incapacity experienced by women during migraine episodes leads to feelings of vulnerability and insecurity about their dependence on others and ability to take care of their children during migraine attacks (Rutberg & Öhrling 2012:332). The participants with children in this study did not report insecurity about their ability to take care of their children during migraine episodes; however, they experienced significant family burden as caregivers. One participant did not have assistance with taking care of her family during migraine episodes, which led to family burden and the feeling of hopelessness. Furthermore, a narrative review revealed studies, which reported that family burden increased consistently with migraine frequency; limitations were noted in caring for and dealing with their children; and there is a burden associated with being a caregiver with migraines (Leonardi & Raggi 2019:6). Most participants in this study withdrew or isolated themselves from their families, and some participants experienced the burden of taking care of their children during migraine attacks. Significant family burden was also reported in the recent Chronic Migraine Epidemiology and Outcomes (CaMEO) study (Estave et al. 2021:1009).

The third sub-theme of Theme 1 focused on fear-avoidance behaviour and lifestyle changes. Fear-avoidance behaviours in relation to migraines are the actions taken to avoid triggering migraine attacks. The participants avoided migraine-triggering factors, through fear-avoidance behaviours, and were cautious about their actions and activities. A comprehensive study conducted in 2021 indicated that despite efforts taken to avoid triggering factors, women with migraines still experienced migraines that were sufficient to interfere with the ability to perform daily activities at work and at home (Connor et al. 2021:4). Malone et al. (2015:540) reported that 88.3% of their study population, which were predominantly female and chronic migraine sufferers, actively avoid migraine triggering factors. The participants in this study feared the onset of migraines while driving, attending important work events, attending social activities, and meeting new people. They also feared the development of a greater intensity of pain. A qualitative study that predominantly consisted of women (90%), included the theme of ‘fear and avoidance’, and the sub-themes included ‘anticipatory anxiety’ and ‘avoidance behaviour’, which was similarly documented in this study (Estave et al. 2021:1009). Anticipatory anxiety is the ‘fear of future attacks and subsequent avoidance of situations thought to trigger attacks’ (Estave et al. 2021). Fear-avoidance behaviours and anticipatory anxiety in women with migraines were substantiated in previous literature (Estave et al. 2021; Palacios-Ceña et al. 2017:9). In this study, anticipatory anxiety was common among most participants. This led them to display fear-avoidance behaviours and adopt drastic changes in their lifestyle since their migraine onset. As a result of the fear of a potential onset of a migraine, the participants postponed or declined work trips or events. Furthermore, most participants required constant access to migraine medication in anticipation of the onset of a migraine.

The fourth sub-theme of Theme 1 is ‘acceptance of migraines’. The inability to accept pain can lead to a reduced quality of life, greater disability, and depression, whereas pain acceptance can lead to a greater pain tolerance in those with chronic pain (Dindo et al. 2015:110). Women with chronic migraine ‘convey resignation at not being able to live completely pain free and they have learnt to accept that they must live with it’ (Palacios-Ceña et al. 2017:9). Women with chronic migraine accepted migraines as a permanent part of their lives, even with treatment and changes to their lifestyle (Banciu & Bouleanu 2018:28). Migraineurs who were able to cope with commitments and obligations around their migraines, were more accepting towards their migraine-related pain (Peters et al. 2005:44). In this study, some participants accepted their migraines as a normal part of their lives. Half of the participants did not allow their migraines to affect them personally at the time of this study because of their duties towards their families, although most participants reported having a history of associated anxiety and depression with their migraines. The participants attempted to endure their migraines even if they were in severe pain, because they were obligated to fulfil their work and family duties. Similarly, women with migraine also attempted to endure their migraine attacks (Estave et al. 2021:9). The participants took comfort in knowing what to expect with their migraines, which allowed them to accept the impact it has on their lives. The literature also revealed that women perceive migraines as a part of themselves because they experienced familiarity with regular attacks (Banciu & Bouleanu 2018). Furthermore, this perception could be based on their recurrent pain episodes or the experiences of migraineurs known by them (Banciu & Bouleanu 2018). One participant felt as though she lost out on life with every day she was incapacitated because of bed rest associated with a migraine and was unable to accept the disability the migraines caused.

Theme 2 focused on the effects migraines had on relationships. Two sub-themes emerged from the data. The first sub-theme described the ‘impact on family and social networks’, which included both strained and supportive relationships. This study found that half of the participants experienced challenges with personal or family relationships. Initially, some participants experienced a lack of understanding from a parent’s or husband’s perspective about the extent of the suffering they experienced. However, over time their personal relationships improved because of their families witnessing their episodes of migraine attacks. Two participants were previously accused of malingering about their pain or making excuses to leave family events when experiencing migraines. A theme encountered among women with chronic migraine was ‘family and work: between understanding and disbelief’, where the participants were accused by their families of exaggerating their migraine-related pain because of disbelief about their pain severity (Palacios-Ceña et al. 2017:9).

Migraines had a significant impact on all the participants’ social lives. A few participants had challenges with their social networks while cancelling social plans. This resulted in strained short-lived relationships. Some participants hid their experience of migraines from family, peers, and work colleagues to avoid being judged. Hence, the participants made excuses to those in their social networks to avoid visiting certain places that triggered their migraines. In a study consisting of 84% women, the social impact of migraines included limitations in social interactions and participation in social events involving loud noises and bright lights (Mannix et al. 2016:6). Participants had difficulty meeting new people socially in anticipation of experiencing a migraine. The participants also described the lack of understanding they encountered in social interactions about their migraine pain severity. Similarly, in social circles, women with chronic migraine experienced certain disbelief when talking about their migraine pain. As a result, they often hid their migraine attacks (Palacios-Ceña et al. 2017:11). Furthermore, Malone et al. (2015:539) reported that nearly three quarters of their study participants’ (92.8% female) social networks did not believe the participants when they mentioned the pain severity of their migraines. Some participants in this study expanded their social circle to a supportive network and support groups that accepted them and assisted them when they experienced migraines. In previous studies, friends, family or colleagues assisted migraineurs when they could not perform tasks (Gibbs et al. 2020:1351; Peters et al. 2005:44). The local literature revealed that among South African university students who attended social events (60% of the population were women), migraineurs left early, drank water, requested medication or isolated themselves (Basdav 2016:3). There were only two participants who did not experience a history of strained relationships in this study and most of the participants have since built more supportive relationships.

The second sub-theme of Theme 2 discussed the ‘impact of migraines on work life’. Migraines affected the participants’ concentration at work. A few participants hid their migraines, were not willing to disclose their migraines to their co-workers and made excuses for ignoring them during a migraine attack. Some participants needed to take leave from work because of being unable to adequately function with a migraine attack. Women were distressed when a migraine developed at work and had trouble coping with the pain as they maintained their work obligations (Connor et al. 2021:4). Sub-themes that were identified among women with migraine were ‘living with fear of not being believed’ and ‘struggling to avoid being doubted’, and this led them to hiding their migraine symptoms from others (Rutberg & Öhrling 2012:333). Furthermore, these women did not perform their best at work when they had a migraine. This periodically resulted in them being unable to fulfil their work obligations, which hindered work opportunities (Rutberg & Öhrling 2012). A few participants in this study also frequently met with co-workers who did not understand the severity of their headaches. Women with chronic migraine also encountered a lack of understanding from their co-workers about the severity of their migraine pain (Palacios-Ceña et al. 2017:9). Most of the participants in this study were allowed to take leave from work for their migraines and had an understanding work environment. Although their migraines caused functional disability, most participants fulfilled their work obligations and deadlines because they could not afford to take leave from work often. Therefore, presenteeism was encountered more frequently than absenteeism in this study.

Theme 3 focused on the ‘approach to self-management of migraines’. Under this theme, two sub-themes emerged from the data. The first sub-theme focused on the ‘resistance to medication’. Side effects were experienced by most participants with their prescribed migraine medication. The side effects experienced were fatigue, drowsiness, sleep disturbances, and depression. The participants preferred to undertake lifestyle changes and holistically manage their migraines, as opposed to relying on pharmacological therapy. The theme ‘desire for a more holistic, collaborate approach to treatment’ was evidenced among the migraineurs (majority of whom were female) and they described the importance of treatment tailored to their needs and their preferences for migraine management (Befus et al. 2019:3). The participants in this study were resistant to taking medication because of its side effects and preferred to use natural alternatives. The theme ‘worrying about the use of medication’ was found among female migraineurs as it raised fears about addiction and the long-term effects (Rutberg & Öhrling 2012:333). In addition, the use of medication for migraines did not restore full function and was accompanied by dizziness, lethargy and reduced concentration. Although prescribed medication provided temporary relief, these women negotiated with themselves before taking medication and felt as if they did not have an alternative treatment (Rutberg & Öhrling 2012). The theme ‘medication as a short-term solution’ was found among the migraineurs (83% female participants) as there was a desire to use non-pharmacological approaches for migraine management for long-term migraine reduction, prevention and contributions to overall health (Befus et al. 2019:4). Furthermore, although pharmacological treatment was found to be effective, it was discontinued because of its side effects such as fatigue, dizziness, nausea and ‘spaciness’ (Befus et al. 2019:4). Women stated that the lingering side effects of the migraine and their medications prolonged their recovery time (Connor et al. 2021:4).

The second sub-theme of Theme 3 related to the ‘factors that alleviated migraines and alternative therapies’. Regarding self-management strategies, most participants preferred alternative, holistic therapies to manage their migraines. The self-management strategies that the participants used to cope with their migraines were sleep, cold showers, ice packs, heat, stretches, exercise, physical activity, Botox, topical analgesics, and medication (migraine packs, migraine mediation, anti-inflammatories, or analgesics) if required. ‘Botox (known chemically as botulinum toxin type A) is a purified neurotoxin complex, which has neuromuscular transmitter blocking effects’ (National Institute for Health and Care Excellence 2012:1). The National Institute for Health and Care Excellence (NICE) recommend the use of Botox for chronic migraine that has not responded to at least three prior pharmacological prophylaxis therapies (Herd et al. 2019:7; National Institute for Health and Care Excellence 2012:1). A systematic review and meta-analysis inclusive of 28 trials revealed that Botox reduced migraine frequency by 2 days per month in chronic migraineurs (Herd et al. 2019:1). To alleviate pain, migraineurs also used breathing exercises, aerobic exercise, neck exercises, biofeedback, behavioural or psychological approaches (Moriarty & Mallick-Searle 2016:30), specific food or drink, compresses (Befus et al. 2019:5), relaxation, rest, sleep (Banciu & Bouleanu 2018:29), and altering their posture (Connor et al. 2021:4). In South Africa, migraine alleviating factors included medication, massage, lying down, exposure to heat or cold, and comforting food (Du Preez 2004:112).

A strong interest in more ‘natural’ migraine management approaches were expressed by migraineurs (Befus et al. 2019:5). The alternative therapies used by the participants in this study included chiropractic, massage, meditation, reflexology, yoga, cupping and acupuncture. The literature supported these findings (Befus et al. 2019:5; Rhee & Harris 2017:97):

Reflexology is an ancient therapeutic treatment which activates the innate healing powers of the body by applying pressure to the specific reflex points on the feet, hands, or ears. Recent evidence suggests that reflexology is a simple, less expensive, and non-invasive method that is effective to regulate the autonomic nervous system activities, coordinate physical responses, alleviate anxiety, and it induces body relaxation. (Marican et al. 2019:514)

Reflexology was also used among other migraineurs for pain relief (Befus et al. 2019:5; Kobza, Lizis & Zięb 2017:217). A study conducted among female migraineurs showed that both reflexology (10 treatments twice per week) and massage (15 treatments thrice per week) effectively reduced the pain intensity, frequency and duration of migraine attacks in women up to 3 months after treatment (Kobza et al. 2017). Massage was also used by other migraineurs for alleviating pain (Befus et al. 2019; Moriarty & Mallick-Searle 2016:30; Rhee & Harris 2017:97).

Acupuncture in Traditional Chinese Medicine is characterised by the insertion of needles into the skin to stimulate acupuncture points on the body, for the treatment of diseases and regulation of the Qi (energy) flow into rebalance (Zhu et al. 2021:2365). A comprehensive Cochrane review of 22 trials (4985 participants) concluded that the available evidence suggests that acupuncture moderately reduces the frequency of migraine attacks, is similarly as effective as treatment with prophylactic drugs, and can be considered a treatment option for migraines (Linde et al. 2016:2). Studies have shown that migraineurs used acupuncture for their migraines (Befus et al. 2019:5; Moriarty & Mallick-Searle 2016:30).

Yoga was found to be a CAM treatment used among migraineurs (Befus et al. 2019:5; Boroujeni et al. 2015:1; Moriarty & Mallick-Searle 2016:30). Yoga is a holistic practice and ‘an empirically evidenced modality in [the] allied healthcare field’ which uses various yoga postures, breathing exercises and meditation (depending on the purpose and type of yoga) for the promotion of physical and mental health (Boroujeni et al. 2015). In female migraineurs who received yoga therapy, after 3 months of intervention there was a significant reduction found in migraine frequency, severity and impact on patients’ lives (Boroujeni et al. 2015).

Another complementary and alternative therapy used by migraineurs was cupping (Alshawish et al. 2021:254; Kaki et al. 2019:105). Cupping is a traditional medicine practice, which uses suction cups on the body to alleviate pain; improve circulation; and balance the immune, nervous, and hormonal systems. Dry cupping involves air suctioning, whereas wet cupping is a bloodletting method, which involves superficial incisions and the removal of subcutaneous blood through suction cups (Alshawish et al. 2021; Kaki et al. 2019).

Chiropractic was the most effective and preferred approach for migraine pain management in this study, despite the participants’ experience with other treatments. The participants stated that chiropractic reduced the severity of their migraines and disability, as well as the duration of suffering. It also reduced the need for pharmacological interventions. Previous South African studies quantitatively documented that chiropractic SMT decreased migraine frequency, severity, and disability and improved the quality of life (Chopdat 2015:vii; Du Preez 2004:132).

## Limitations

The sample was restricted to only the participants residing in the eThekwini region of KwaZulu-Natal in South Africa. Despite it being a small sample, information richness was still obtained. The study utilised purposive sampling; participant recruitment, therefore, focused only on a few regions in the eThekwini Municipality District. As a result of the COVID-19 pandemic, most of the interviews were conducted virtually. Although rich information was captured through video calls, face-to-face interviews would have been preferred to extract rich data.

## Recommendations

Future studies should include more qualitative research studies on the psychosocial impact of migraines among the female population. There is also a need for more qualitative literature to highlight the efficacy of CAM for migraines in South Africa.

## Conclusion

This study provided insight into the psychosocial impact of migraines on women and their experience of complementary and alternative therapies for migraine pain management. Our findings emphasised the burden of migraines, its impact on the quality of life in women, and coping strategies used to alleviate pain. Migraines changed the participants’ personalities and lifestyles as they displayed anticipatory anxiety and fear-avoidance behaviours to circumvent triggering their migraines. The pertinent psychological effects included depression, anxiety, anger, frustration, irritability and hopelessness when experiencing migraines. Participants experienced strained relationships with co-workers, family and social networks who did not understand the severity of their migraines. Although the migraines caused functional disability, most of the participants fulfilled their work obligations because they could not frequently afford to take leave from work. The self-management strategies for migraine-related pain included sleep, cold showers, ice packs, heat, stretches, exercise, physical therapy, Botox, and analgesics if required. The alternative and holistic therapies that were sought to alleviate migraine symptoms included chiropractic, massage, meditation, reflexology, yoga, cupping and acupuncture. The study shed light on important implications for clinical practice. The knowledge of participants’ experiences of migraines brings awareness to the impact of their suffering and highlights the importance of patient-centred care, which can contribute to more efficient doctor–patient relationships and more effective care that is tailored to their needs and preferences. This study builds on the body of knowledge of treatment approaches for migraine management in clinical practice. Migraine management is patient-specific, and awareness of the effectiveness of CAM therapies for women may be beneficial for healthcare providers seeking a multidisciplinary approach to migraine management. This study is of relevance to the psychosocial burden of migraines and the implementation of beneficial treatment strategies to improve treatment outcomes for migraines in the predominantly affected female population.
